# Enhancing coping skills through brief interventions during cancer therapy – a quasi-experimental clinical pilot study

**DOI:** 10.3389/fpsyg.2023.1253423

**Published:** 2023-09-07

**Authors:** Norbert Gelse, Daniela Bodschwinna, Marc N. Jarczok, Magdalena Wanner, Madeleine Volz, Regine Mayer-Steinacker, Jens Huober, Harald Gündel, Klaus Hönig

**Affiliations:** ^1^Department of Psychosomatic Medicine and Psychotherapy, University Ulm Medical Center, Ulm, Germany; ^2^Comprehensive Cancer Center Ulm, University Ulm Medical Center, Ulm, Germany; ^3^Department of Internal Medicine III, University Ulm Medical Center, Ulm, Germany; ^4^Department of Gynecology and Obstetrics, University Ulm Medical Center, Ulm, Germany; ^5^Cantonal Hospital, Breast Center St. Gallen, St. Gallen, Switzerland

**Keywords:** coping with cancer, hypnosis in outpatient settings, resource activation, psycho-oncology, self-hypnosis, cognitive-behavioral interventions, potential of brief interventions, feasibility of brief interventions

## Abstract

**Introduction:**

Psycho-oncological interventions can reduce distress by activating individual resources and enhancing coping skills. Since medical cancer treatment is performed increasingly in outpatient settings, there is a growing need for evidence-based and brief interventions to be integrated seamlessly into these treatment procedures. The aim of the present pilot study is to examine the feasibility of brief interventions to cope with illness in this area.

**Methods:**

A single center quasi-experimental design was developed in oncological outpatients at the University Medical Center Ulm, Germany, including *N* = 60 individuals with cancer undergoing chemotherapy or immunotherapy. The intervention group (IG) consisted of *N* = 40 participants. These were assigned to either cognitive behavioral interventions (CBI) or hypnotherapeutic interventions (HTI). The interventions each comprised three individual one-hour sessions. In addition, a waiting control group (WCG of *N* = 20) was set up, receiving care-as-usual. Primary outcomes were feasibility measures such as recruitment rates, participant retention rates, and complete data rates. Clinical results were discussed for the feasibility of a comprehensive efficacy study.

**Results:**

The recruitment and completion rates illustrate demand and acceptance of the offer. Of the 208 individuals with cancer offered to participate in the study, 77 were interested in enrolling. This rate of 37% roughly corresponds to the use of psycho-oncological services in general. 17 individuals (22%) withdraw from participation before the intervention began due to severe deterioration in their disease. Once started, all 40 individuals of the IG (100%) completed the intervention, and 17 individuals of the WCG (85%) completed the accompanying questionnaires. Tentative results on clinical outcomes indicate that brief interventions on resource activation could have lasting effects on well-being and stress management.

**Discussion:**

With this feasibility study, we aimed to explore the potential of brief interventions such as hypnotherapeutic and cognitive-behavioral approaches in psycho-oncology as an integral part of oncology day care. Even with a small number of participants results seem to indicate that the study design and brief interventions such as those presented can offer a low-threshold service that can be seamlessly integrated into oncological therapy. Given the promising results of this pilot study, we propose a full RCT on the effectiveness of such a brief intervention program.

**Clinical trial registration:**

https://www.drks.de, German Trials Register (DRKS00019095).

## Introduction

Subsyndromal stress, such as distress, anxiety, fear of progression and depression, occurs in over 50% of individuals with cancer (Mehnert et al., 2018). About a third of these receive psychological support during a hospital stay (Weis et al., 2018). This support should be accessible early and complement clinical therapy (Holland, 2003; Holland and Weiss, 2008). Early assessment and distress screening should lead to timely treatment of mental stress, which can improve medical care (Riba et al., 2019). International guideline programs, including the National Cancer Plan in Germany, recommend making it mandatory to offer psycho-oncological services if necessary. They should be an integral part of oncology care not only for inpatients, but also in day care and outpatient sectors of the healthcare system (Fann et al., 2012; Grassi and Watson, 2012; Bergelt et al., 2016; Rosenberger, 2018; AWMF e.V., 2023).

In face of a constant reduction in inpatient stays and an increasing importance of day clinic care, the need for effective and validated short-term interventions is likewise growing (Carlson and Bultz, 2004; Abrams et al., 2018; Blümel et al., 2020; Schuit et al., 2021).

Resource-oriented methods seem particularly suitable here. Due to the high psychological and physical symptom burden of the individuals and the associated, sometimes pronounced psychological defense mechanisms, more indirect and experience-based approaches could offer effective relief and support. Even the disclosure of a cancer diagnosis is often shocking and frightening for those affected, coupled with uncertainty about treatment options, their consequences, a possible prognosis and the fear of progression or recurrence. Life suddenly and unexpectedly seems to be “out of control.” Potentially available psychological resources appear limited or unattainable. Resource-activating interventions can help regulate emotions, build resilience, and encourage a more problem-solving attitude by focusing on the healthy parts of an individual (Gassmann and Grawe, 2006; Flückiger et al., 2009; Groß et al., 2012).

Since there are hardly any studies on the feasibility and effectiveness of brief interventions using resource-activation in oncological outpatient settings, we wanted to start filling this empirical research gap and encourage further controlled studies. Therefore, we developed a brief intervention program, HypRa (Hypnosystemic Resource activation), which should be low-threshold and seamlessly integrated into a clinical oncological outpatient setting.

Based on the concept of resource activation, HypRa aims to help individuals with cancer find perspectives on well-being and solutions to cope with stress by activating their abilities. The focus was on the potential and applicability of brief hypnotherapeutic interventions, particularly self-hypnosis, and cognitive behavioral interventions to strengthen coping skills in the supportive care of individuals with cancer during clinical treatment.

There are good arguments related to resource activation for both types of intervention. For hypnotherapeutic interventions, there is also specific evidence that these interventions could be effective quickly. Many studies already report positive effects in hypnotherapeutic treatment of symptoms in individuals with cancer (Montgomery et al., 2013; Cramer et al., 2015; Carlson et al., 2018). These studies typically focus on distress associated with medical procedures (Schnur et al., 2008), nausea and vomiting (Marchioro et al., 2000; Montgomery et al., 2007), hot flashes (Elkins et al., 2008), and pain (Spiegel, 1985; Elkins et al., 2012; Kravits, 2013; Nakandala, 2021). According to the results of these studies, there are some indications that just a few sessions are enough to bring about lasting relief from physical symptoms. For example, treating hot flashes in individuals with breast cancer using self-hypnosis training with five weekly sessions showed a 69% reduction in hot flashes on average from baseline and reduced disruption to daily activities, sleep, anxiety, and depression (Elkins et al., 2008).

In a meta-analysis of randomized trials on hypnosis to manage distress related to medical procedures, there were indications that approximately 82% of individuals who receive hypnosis live/pre or while undergoing medical procedures exhibit lower levels of emotional distress relative to individuals in a control condition, with a larger effect size for children compared to adults (Schnur et al., 2008).

For interventions to reduce anxiety and stress, Carlson et al., 2018 refer to a meta-analysis by Chen et al. (2017) showing that hypnosis had significant immediate and lasting effects on anxiety in individuals with cancer. Again, larger effect sizes were found in the pediatric group, and therapist-administered hypnosis was more effective than self-hypnosis. However, there is no indication here of the form in which self-hypnosis was learned and used.

While hypnotherapeutic interventions in oncology are not yet widespread, cognitive-behavioral therapeutic interventions can now be regarded as a kind of gold standard in supportive psycho-oncological treatment for emotional relief and stabilization as well as for better coping with cancer (Moorey and Greer, 2011; de Vries and Stiefel, 2014). These interventions typically focus on methods based on mindfulness, self-care and communication skills (Tatrow and Montgomery, 2006; Daniels, 2015; Ye et al., 2018; Getu et al., 2021).

Investigating the feasibility of these interventions – here with only 3 sessions – in a clinical environment is a relatively new area of research. In order to make the offer as low-threshold as possible for the individual and to enable easy integration into oncological therapy, the study design was structured in such a way that, for example, short-term adjustments were possible if appointments had to be postponed. It should fit well into the setting of a day clinic, which means that the medical and nursing team supports the offer by providing premises and with administrative or coordinating questions. The work processes should be disturbed as little as possible. In addition, reference should be made to the information material for recruiting participants on site. The interventions themselves were not designed as group sessions but as individual sessions.

We were initially interested in how great the interest and the acceptance of individuals with cancer would be in the psycho-oncological offer accompanying their oncological therapy.

Due to the small sample size, we combined the two interventions for the analysis in an intervention group (IG) in a first step. We hoped to gain insights into the potential of the interventions overall and, if necessary, also in a comparison of the two approaches for further investigations. However, conclusions on clinical efficacy should be drawn with extreme caution and interpreted as preliminary. Rather, they should give reason to be examined in a larger randomized study.

## Materials and methods

The present study design is a 3-arm quasi-experimental pilot study. The protocol was approved by the Institutional Review Board of Ulm University (No. 431/16, 08/02/2017) and registered at the German Trials Register (DRKS00019095). The study complied with the Declaration of Helsinki, the Guideline for Good Clinical Practice, and local regulatory requirements. All participants provided written informed consent prior to inclusion.

### Participants

Participants were recruited and enrolled at the Medical Oncological Outpatient Clinic of the Department for Internal Medicine I (MOT) and the Interdisciplinary Oncological Outpatient Clinic of the Clinic for Gynecology and Obstetrics (IOT) at the University Ulm Medical Center, Ulm, Germany. At the outpatient clinics, individuals with gastrointestinal, lung, leukemia or breast and gynecological cancers are treated with chemotherapy or immunotherapy in all phases of the disease. Exclusion criteria were limitations in mobility, hearing and communication abilities, and participation in other psychotherapeutic treatments.

### Study design

Recruitment and enrollment took place from September 2017 to March 2019. Individuals being treated in the day clinics were offered the opportunity to take part in a therapy-accompanying psycho-oncological study on resource activation, regardless of the diagnosis, the time of diagnosis and the duration of the cancer disease. They were informed that this offer is aimed at strengthening their own stress management skills and consists of 3 individual sessions over a period of approx. 6 weeks and 3 questionnaires over a period of approx. 5 months. If individuals were interested and gave consent to participate in the study, they were assigned to one of two intervention arms, cognitive behavioral intervention (CBI) or hypnotherapeutic intervention (HTI). Taking into account the clinical environment, this quasi-experimental approach was based on the available places in each intervention arm at any given time. Participants then received three individual one-hour sessions every two weeks, including homework between sessions. Questionnaires were used at three measurement time points (T0: pre-test as baseline value before the first session; T1: post-test after the end of the last session, T2: follow-up three months after the last session). In addition, a waiting control group was set up (WCG of *N* = 20). If interested, the WCG individuals had the option of later being included in the intervention program. In the meantime, they received care as usual (CAU). They were asked to fill out the T0 and T1 questionnaires at two points (six weeks apart). This methodological approach has been recommended for ethical reasons.

### Measures

Because this pilot-study is focusing on feasibility, the primary outcome measures were the total number of individuals contacted, relative interest in the service and participation, completion of participation for the intervention group (IG), and questionnaire completion by responding participants of the waiting control group (WCG). With caution, satisfaction with the intervention can be indirectly inferred from the tentative trends on changes in resource activation and stress coping skills as measured with the questionnaires at T1 and T2. All participants of the study received a set of standardized questionnaires on resource activation as measured by the Bern Resource Inventory (BRI), and individual stress management abilities as measured by the Inventory of perceived Stress Management Skills (ISBF). The BRI is a self-report questionnaire covering eight categories of personal resources (Trösken and Grawe, 2004). For practical reasons, we have selected the items on well-being, personal strengths, and former coping with crisis. The ISBF covers perceived stress management skills like cognitive strategies, use of social support, relaxation strategies, anger regulation, and perception of bodily tension (Wirtz et al., 2013).

### Interventions

Both interventions, HTI and CBI, started with psychoeducation to explain the psychophysiological mechanisms of individual stress experiences and how these can be modulated through activating personal resources (Lazarus, 1974; Schneiderman et al., 2005).

In the first session of CBI, a multifactorial model was introduced to the individuals to promote a better understanding of factors contributing to psychological distress and clinical symptoms or providing resilience. The model included biological factors (e.g., the sensation of pain, autonomic bodily reactions to stress), psychological factors (e.g., thoughts, emotions and behavior connected to illness, self-affirmation, social skills, enjoyment) and social factors (e.g., social support system, working ability, participation, social security) and was then adapted to the individual life situation (Kaluza, 2018).

In the second CBI session, guided mindfulness exercises were instructed, for example, mindful breathing, smelling, observing or experiencing bodily sensations (Ledesma and Kumano, 2009). Participants were encouraged to include mindfulness practice in their everyday life in-between sessions. In the third session, self-care and social skills methods were targeted, including education and exercises on beneficial communication techniques and self-management in social situations (Beck, 2011). Further, prioritizing own needs by establishing regular pleasant activities and reducing the personal load in everyday life was discussed.

Following the psychoeducation already mentioned above, the hypnotherapeutic intervention (HTI) began with an introduction to a first trance experience, e.g., an imaginative journey to a personal “place of well-being” combined with a guided imagination promoting indirect access to beneficial emotional experiences (Bongartz and Bongartz, 2009). In order to enable low-threshold access to resource experiences in trance, items from the BER questionnaire were used (Wolf and Bongartz, 2009). Representative figures (e.g., tensing and relaxing of the muscles, outflow of a dam, rocks in the surf, seagulls on the sea in the wind, flowers in spring, calm in the valley) metaphorically symbolize resources such as power, release, safety, trust, hope, and clarity and allow individuals easier access to their own emotional resources. Individuals were then asked to rate these experiences as personal resources for their everyday lives. Especially when internal resources do not currently appear accessible, clinical experience shows that symbolizations of potential resources can be introduced, utilized and thus integrated into a person’s abilities (Hönig, 2017; Revenstorf, 2017).

After the first HTI session, participants were encouraged to continue practicing the trance experience using a pre-recorded take-home audio file with imaginations about well-being, safety, trust, and hope. In the second session, based on the personal resources mentioned, an individual trance story was developed and practiced as self-hypnosis under guidance. This story was recorded during the session to continue practicing self-hypnosis at home. In the third session, the self-hypnosis experiences were evaluated and modified, if necessary, as a kind of tool for further individual stress management, always and everywhere available, if required. For example, individuals can use it before surgery, during radiation therapy, in a treatment room, in the hospital bed, or at night when they are having trouble falling asleep (Montgomery et al., 2013).

In both intervention arms, participants were given homework between sessions, including excerpts from the BRI (see above) to reflect personal resources, strengths, and abilities to cope with stress and crisis. Reflection thoughts and diary entries were discussed for further treatment. At the end of the third session, reported experiences and insights during the interventions were summarized. Finally, the T1 questionnaire was handed out, and participants were informed that another questionnaire (T2) will be sent approximately three months after this session. In addition, information was provided about other options for counseling by the inpatient psycho-oncological service or a cancer counseling center after participating in the study.

### Statistical analysis

Baseline values of all variables were compared as means and standard deviations (SD) or Count (N) and Percentages (%) between groups using two-sided t-tests or Wilcoxon rank test (see Supplementary Table S2).

Linear mixed-effects regression models with random intercepts and variance type “identity” were fitted using STATA^©^ 15.1 (STATA Corp, College Station, TX, USA). Time was on level one, and the individual person was on level two. Per outcome, a total of four models were calculated. First, a population mean model with no covariates. The second model additionally included main effects (fixed effect part), the third model additionally included a random intercept of time (random effect part), and the fourth model additionally included a two-way interaction between the groups (IG vs. WCG) and time (T0 vs. T1 vs. T2) in the fixed effect part.

*Post hoc* analysis were conducted by calculating pairwise comparisons of the average predicted probability of the observed outcome per timepoint conditional on the group assignment (i.e., marginal means with group contrasts comparing time points) with groups.

Diagnosis (Gynecological tumors vs. Gastrointestinal tumors vs. other) and disease duration (month) were included in the models as covariates in the fixed effect part. Significance levels were set to *p* < 0.05 to compare models using Chi^2^. Marginal means were estimated and plotted at covariate averages at fixed values for group and time interaction.

## Results

With *proof of concept as primary outcome*, we looked at recruitment rates, participant retention rates, and full data rates. Of the 208 individuals with cancer approached, 77 showed interest in participating in the study. This corresponds to a recruitment rate of 37%. Even before the start of the first intervention session, 17 individuals had to withdraw from participation due to a worsening of their disease. All 40 individuals of the IG (100%) completed the intervention, and 17 individuals of the WCG (85%) completed the accompanying questionnaires.

The sample of *N* = 60 participants who completed the study was distributed as follows: 13 male (22%), 47 female (78%); mean age 55.87 years (SD = 10.83); oncological diseases: breast or gynecological 27 (45%), gastrointestinal 18 (30%), other 15 (25%); mean duration of disease was 21.88 month, ranging from 1 month to 243 months (SD 42.09); mean duration median = 8 months; initial diagnosis 38 (63%), recurrence 22 (37%) – see Supplementary Table S1.

After completion of the respective intervention phase, i.e., after about 6 weeks plus a follow-up after 3 months, 146 observations from 58 individuals could be evaluated. In the follow-up, there were incomplete questionnaires in a total of 6 in the individuals in the intervention groups and in a total of 5 in the WCG – see Consort Flow Diagram in Supplementary Table S3.

No adverse events were observed due to the intervention.

Preliminary findings on *secondary clinical outcomes* were assessed by changes in resource activation and stress management skills after three individual sessions comparing intervention (IG) versus waiting state (WCG) as measured by standardized questionnaires (BRI and ISBF).

**Figure 1:**
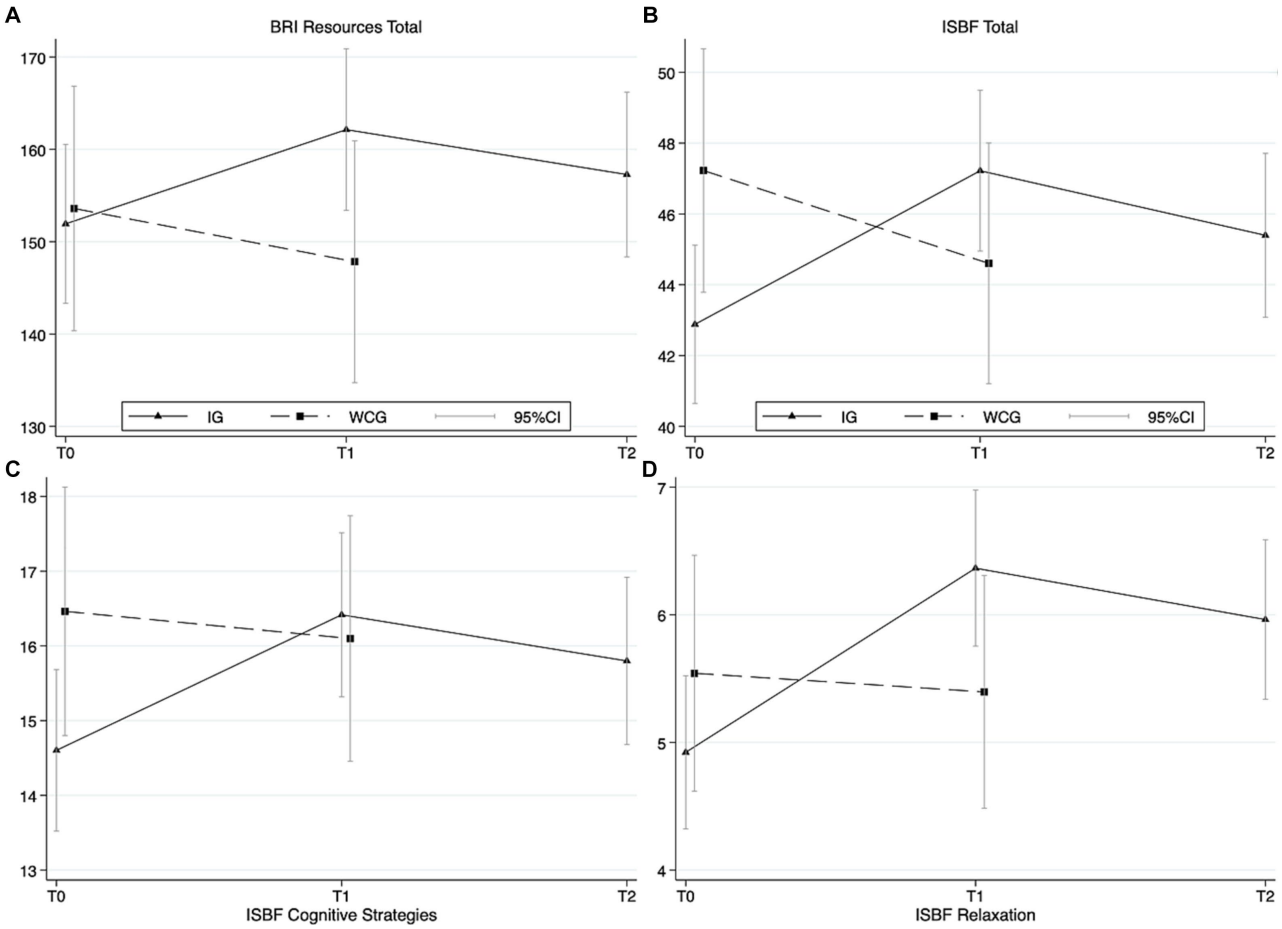
Results from linear mixed-effects regression models (marginal means) for resources and stress management skills **(A)** BRI Resources (Total Score), **(B)** ISBF (Inventory for Stress Management Skills) total score, **(C)** ISBF Cognitive Strategies, and **(D)** ISBF Relaxation Techniques. Models were adjusted for diagnosis and duration of disease.

The statistical models encountered no convergence issues for any of the outcomes. Changes in resource activation and stress management capabilities were observed. The systematic model comparison revealed model 4 as the favored model for the outcomes of BRI Total score, ISBF Total Score, Cognitive Strategies and Relaxation Techniques (Model 4 = group × time interaction), indicating systematic trajectory differences for the groups over time (see Supplementary Table S2 for detailed model comparison, see also Figure 1).

Concerning activating their resources, the intervention group (IG) showed higher scores at T1 compared to T0 (*p* ≤ 0.05), measured by the BRI (see contrasts in Table 1). The total score for stress management skills as measured by ISBF increased for IG from T0 to T1 (*p* ≤ 0.001). The stress management subscore for relaxation skills increased at T1 for IG (*p* ≤ 0.001). For WCG, small changes in terms of a decrease in resource activation and stress management skills were observed from T0 to T1 (see Table 1).

**Table 1:** Contrasts within predictors.

Variables	Comparison within IG			Comparison within WCG
	T1 vs T0	T2 vs T0	T2 vs T1	T1 vs T0
BRI Resources Total	10.22**	5.28	−4.93	−5.78
ISBF Total	4.35***	2.45**	−1.89**	−2.63*
ISBF Cognitive Strategies	1.81***	1.20***	−0.62	−0.36
ISBF Relaxation	1.45***	1.03***	−0.42	−0.15

All results were independent of the underlying diagnosis and the disease duration across all models. *p ≤ 0.05; **p ≤ 0.01; ***p ≤ 0.001.

## Discussion

With this feasibility study, we aimed to explore the potential of brief interventions such as hypnotherapeutic and cognitive behavioral approaches in psycho-oncology as an integral part of oncology day care. The focus was on the design of a practical program on resource activation for individuals with cancer.

### Key findings

With regards to the feasibility findings as primary outcomes the recruitment and completion rates illustrate demand and acceptance of the offer. Of the 208 individuals with cancer offered to participate in the study, 77 were interested in enrolling. This rate of 37% roughly corresponds to the use of psycho-oncological services in general (Weis et al., 2018). 17 individuals (22%) withdraw from participation before the intervention began due to severe deterioration in their disease. Once started, all 40 individuals of the IG (100%) completed the intervention, and 17 individuals of the WCG (85%) completed the accompanying questionnaires. The preliminary trends on changes in resource activation and stress management skills can also indirectly allow preliminary conclusions to be drawn about satisfaction with the intervention (see below).

Concerning the secondary outcomes, due to the small sample size, we have to be cautious in interpreting the clinical results. Measured with standardized clinical questionnaires, the brief interventions applied – both cognitive behavioral and hypnotherapeutic – tentatively show positive effects after only three individual sessions on resource activation and stress management skills, probably even up to 3 months after intervention. If this trend is confirmed, effective psychological support could be offered with these short interventions for individuals with cancer, especially in this vulnerable phase of therapy. Only a few empirical findings show such a possible effectiveness after short-term interventions as assumed here. Based on a systematic review of the effects of psycho-oncological interventions on emotional stress, anxiety and depression, and quality of life, short-term effects of relaxation training were identified. Larger effects were found for the moderator variable duration of intervention, while longer interventions produced more lasting effects (Faller et al., 2013). For brief interventions in particular, positive effects were reported by psychosomatic-psychiatric liaison services, such as those offered in general hospitals for the initial treatment of psychological comorbidities such as anxiety and depression (Stein et al., 2020). However, an indication is required to take advantage of this offer, which may not (yet) exist in the case of subsyndromal stress in individuals with cancer.

In psycho-oncological settings, some combined approaches of hypnotherapeutic and cognitive behavioral interventions have already been tested. The results are promising and underline our suggestion for a combination, as hypnosis has been shown to enhance the efficacy and benefits of other therapeutic approaches, such as cognitive behavioral therapy (Kirsch et al., 1995; Bryant et al., 2005; Schnur et al., 2009; Eason, 2013; Mendoza et al., 2017; Temple, 2017). For our case, we are encouraged to develop the HypRa program further using a three (or four) session design based on the resource activation principle as described in this pilot-study.

### Methodological limitations

Although the relatively small study population does not allow any conclusions to be drawn about the outcomes of the intervention, we see the great potential of these interventions and the feasibility of this study for a larger RCT. However, what we have to consider concerning psycho-oncological studies the following can be generally stated: On the one hand, different psycho-oncological treatment options are available to individuals with cancer. But the burden of the course of the disease and the associated limitations may preoccupy the person entirely. Accordingly, they are often elsewhere with their thoughts, and they may also use very different resources to cope with cancer problems (Traeger et al., 2012). This may also have an impact on the willingness to participate in a study.

### Recruitment

We must point out that participants in this pilot-study were not recruited according to their level of distress, as measured by a standardized psycho-oncological screening, but according to their personal preferences for participation or non-participation. With regard to the two intervention arms to which the participants were assigned, the overall offer was positioned as psychosocial support during oncological therapy, so that comparable expectations of the benefit of the program can be assumed for both interventions. According to the information in the questionnaires, the participants did not use any other psychotherapeutic support outside the clinic during the study.

Overall, 37 percent of all individuals approached were interested in participating. The most common reasons given by the individuals not taking up this offer were: “*I do not need it because I have good social or emotional support (partner, family, spiritual beliefs)*,” *I’m not that bad, maybe I’ll come back to that later*” or “*I have reservations about psychotherapy*.” These attitudes are relatively representative of clinical reality and confirm findings from other studies regarding psycho-oncological support (Clover et al., 2015; Weis et al., 2018; Pichler et al., 2022).

### Care as usual from multi-disciplinary teams

In the clinical environment, as in the outpatient day clinic, the multi-disciplinary team of medical therapists and professional oncology nurses is usually one of the most important supporting factors. Multi-disciplinary teams (MDT) have been established in many oncology centers to ensure a coordinated, professionally coordinated therapy regimen, treat somatic side effects and provide lifestyle advice. The benefit of these multi-disciplinary-teams for individuals with cancer and the treating team itself is increasingly being studied and scientifically validated (Taylor et al., 2013; Taberna et al., 2020). The oncological outpatient departments of the University Ulm Medical Center, where the present pilot-study was carried out, also work according to these goals. For this study, we refer to this support as standard care or care as usual (CAU). In addition, psycho-oncological care with 1–3 sessions is optionally possible via a psycho-oncological consultation-liaison service (CLS). The structure and results of this feasibility study therefore not only reflect everyday clinical practice, but can also confirm the importance of integrated professional psycho-oncological offers.

### Summary and outlook

With this feasibility study, we aimed to explore the potential of brief interventions such as hypnotherapeutic and cognitive-behavioral approaches in psycho-oncology as an integral part of oncology day care. Preliminary results seem to indicate that the study design and brief interventions such as those presented can offer a low-threshold service that can be seamlessly integrated into oncological therapy. Considering the clinical environment, we designed a concept of brief interventions that could be applied as a standardized structured program and, simultaneously, individualized for the participants to provide them the best possible psycho-oncological support for their emotional well-being during oncological therapy. Instead of symptom-specific interventions, the focus was on developing and applying general resource-activating methods to strengthen individual coping skills. Given the promising results with all limitations and the feasibility documented in this pilot study, we are encouraged to initiate a prospective full RCT on the effectiveness of the presented brief intervention program.

## Data availability statement

The original contributions presented in the study are included in the article/Supplementary material, further inquiries can be directed to the corresponding author.

## Ethics statement

The studies involving humans were approved by Institutional Review Board of Ulm University. The studies were conducted in accordance with the local legislation and institutional requirements. The participants provided their written informed consent to participate in this study.

## Author contributions

NG: Conceptualization, Validation, Writing – original draft, Writing – review & editing. DB: Project administration, Writing – review & editing. MJ: Data curation, Formal analysis, Software, Visualization, Writing – review & editing. MW: Project administration, Writing – review & editing. MV: Project administration, Writing – original draft, Writing – review & editing. RM-S: Resources, Writing – review & editing. JH: Resources, Writing – review & editing. HG: Resources, Writing – review & editing. KH: Conceptualization, Methodology, Resources, Validation, Writing – review & editing.

## Conflict of interest

The authors declare that the research was conducted in the absence of any commercial or financial relationships that could be construed as a potential conflict of interest.

## Publisher’s note

All claims expressed in this article are solely those of the authors and do not necessarily represent those of their affiliated organizations, or those of the publisher, the editors and the reviewers. Any product that may be evaluated in this article, or claim that may be made by its manufacturer, is not guaranteed or endorsed by the publisher.

## Supplementary material

The Supplementary material for this article can be found online at: https://www.frontiersin.org/articles/10.3389/fpsyg.2023.1253423/full#supplementary-material
