# Supplementary material for: Enhancing coping skills through brief interventions during cancer therapy – a quasi-experimental clinical pilot study
Source: Front Psychol. 2023 Sep 7;14:1253423. doi: 10.3389/fpsyg.2023.1253423 (PMC10513768; doi:10.3389/fpsyg.2023.1253423)
Supplement: Supplementary file 1 [file Table_1.pdf]

Supplemental TABLE S1 Descriptive data of the total sample and the individual samples

Variable	WCG (n=20)		IG (n=40)		Total (N=60)	
**Sex**						
Male	6	10.0%	7	17.5%	13	22.0%
Female	14	23.0%	33	82.5%	47	78.0%
**Age**						
M (SD)	59.8 (9.4)		53.9 (11.1)		55.9 (10.8)	
Min, Max	[42; 79]		[29; 76]		[29; 79]	
**Diagnosis**						
Breast cancer and gynecological tumor diseases	7	12.0%	20	50.0%	27	45.0%
Gastrointestinal tumors	6	10.0%	13	8.0%	18	30.0%
Other	7	12.0%	8	10.0%	15	25.0%
**Disease duration**[a]						
M (SD)	16.8 (18.9)		24.4 (49.9)		21.9 (42.1)	
Min, Max	[2; 61]		[1; 243]		[1; 243]	
Md					8	
**Disease stage**						
Initial diagnosis	15	25.0%	23	18.0%	38	63.0%
Recurrence	5	8.0%	17	15.0%	22	37.0%
**Metastasis**						
Scattering	12	20.0%	18	17.0%	30	50.0%
No scattering	8	13.0%	22	17.0%	30	50.0%

Annotation: [a]duration in months
