# Supplementary material for: Enhancing coping skills through brief interventions during cancer therapy – a quasi-experimental clinical pilot study
Source: Front Psychol. 2023 Sep 7;14:1253423. doi: 10.3389/fpsyg.2023.1253423 (PMC10513768; doi:10.3389/fpsyg.2023.1253423)
Supplement: Supplementary file 2 [file Table_2.pdf]

Supplemental TABLE S2 Model comparison N=146 Observations from 58 Individuals

Variables	Obs	Model Number	Model specification	Fixed Effects added	Random effects		Model fit				LRT Test against nested		
					Subjects (ID)	Item (Day)	AIC	BIC	LL	df	df	χ^2	Prob > χ^2
BRI Resources Total	146 (min. 1, avg 2.5 max 3)	1	RE only	-	intercepts	-	1334.92	1343.87	-664.46	3.00	-	-	-
		2	M1 + FE main effects	Group + Day	intercepts	-	1339.73	1363.60	-661.87	8.00	5.00	5.18	0.39
		3	M2 + RE	-	intercepts	intercepts	1341.07	1367.92	-661.54	9.00	1.00	0.66	0.42
		**4**	**M3 + Interaction**	**Group X Time**	**intercepts**	**intercepts**	**1335.64**	**1368.46**	**-656.82**	**11.00**	**3.00**	**10.09**	**0.02**
ISBF Total	146 (min. 1, avg 2.5 max 3)	1	RE only	-	intercepts	-	956.85	965.80	-475.42	3.00	-	-	-
		2	M1 + FE main effects	Group + Day	intercepts	-	958.23	982.10	-471.12	8.00	5.00	8.64	0.13
		3	M2 + RE	-	intercepts	intercepts	959.54	986.39	-470.77	9.00	1.00	0.70	0.40
		**4**	**M3 + Interaction**	**Group X Time**	**intercepts**	**intercepts**	**940.06**	**972.88**	**-459.03**	**11.00**	**3.00**	**24.17**	**<0.001**
ISBF Cognitive Strategies	146 (min. 1, avg 2.5 max 3)	1	RE only	-	intercepts	-	737.97	746.92	-365.98	3.00	-	-	-
		2	M1 + FE main effects	Group + Day	intercepts	-	737.31	761.18	-360.66	8.00	5.00	10.65	0.06
		3	M2 + RE	-	intercepts	intercepts	739.31	766.16	-360.66	9.00	1.00	0.00	1.00
		**4**	**M3 + Interaction**	**Group X Time**	**intercepts**	**intercepts**	**731.77**	**764.59**	**-354.89**	**11.00**	**3.00**	**11.54**	**0.009**
ISBF Relaxation	146 (min. 1, avg 2.5 max 3)	1	RE only	-	intercepts	-	584.24	593.19	-289.12	3.00	-	-	-
		2	M1 + FE main effects	Group + Day	intercepts	-	581.70	605.57	-282.85	8.00	5.00	12.55	0.03
		3	M2 + RE	-	intercepts	intercepts	583.70	610.55	-282.85	9.00	1.00	0.00	1.00
			**M3 Interaction**	**Group X Time**	**intercepts**	**intercepts**	**569.33**	**602.15**	**-273.67**	**11.00**	**3.00**	**18.37**	**<0.001**

Model 2-4 were adjusted for diagnosis and duration of disease

AIC – Aikake Information Criterion
BIC – Bayesian Information Criterion
LL – LogLikelihood
df – degrees of freedom
LRT – Likelihood Ratio Test
X2 – Chi-square

Model Specification – the current model and what it includes. In the above example this refers to the interactions that have been added. Researchers may choose the labels based on variables that have been added (e.g. subject variable X, item variable X) or a different label that summarises in some consistent way how a model is specified and how complex it is.

Model Name – A short-hand to refer to the larger / more complex model that has been created. In the above example the model name has been derived from the addition of different variables. If researchers are testing successive nested models they may simply refer here to 'Model 1', 'Model 2', 'Model 3' etc.

Nested / simpler model – the model against which the current, more complex one is being tested, using the Model Name as a label.

Fixed effects added – which fixed effect / predictor variables have been added in order for a model comparison to take place (against the nested model).

Random Effects – the random effect structure included in the model, identified by column names for the groupings added as random effects (in this case, Subjects and Items) and whether these were intercepts, or intercepts and slopes for specific fixed effects. In this example, random intercepts have been fit for subjects and items. Where these are unchanged in subsequent a models there is a " in the table cell. For models where slopes are fit for fixed effects, authors could enter text such as 'Slopes for ItemVariable 1' etc.

Model Fit – column names that provide information on aspects of model fit, depending on which variables a researcher is choosing to use (e.g. AIC, BIC, Log Likelihood, R^2 etc.)

LRT Test against nested - results of a Likelihood Ratio Test for the current model against the nested model.
