# Supplementary figures and images for: Enhancing coping skills through brief interventions during cancer therapy – a quasi-experimental clinical pilot study
Source: Front Psychol. 2023 Sep 7;14:1253423. doi: 10.3389/fpsyg.2023.1253423 (PMC10513768; doi:10.3389/fpsyg.2023.1253423)

# CONSORT 2010 Flow Diagram – HypRa – Enhancing Coping Skills

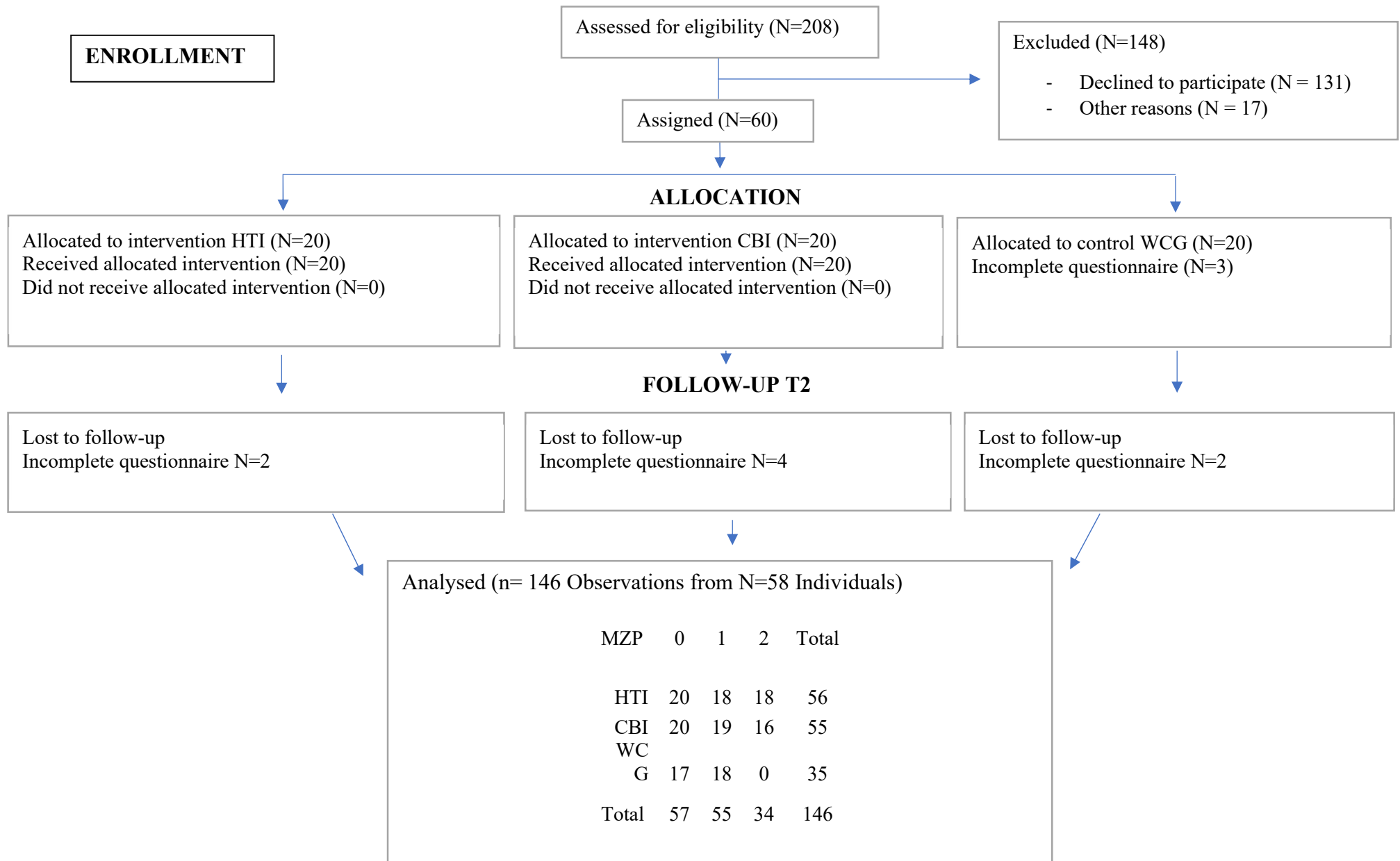

Supplement: Supplementary file 3 [file Table_3.pdf]
